# The Role of Type and Source of Uncertainty on the Processing of Climate Models Projections

**DOI:** 10.3389/fpsyg.2018.00403

**Published:** 2018-03-27

**Authors:** Daniel M. Benjamin, David V. Budescu

**Affiliations:** ^1^Biomedical Ethics Unit, Department of Social Studies of Medicine, McGill University, Montreal, QC, Canada; ^2^Department of Psychology, Fordham University, New York, NY, United States

**Keywords:** sources of uncertainty, conflict, imprecision, climate change, global warming, forecasting, ambiguity, vagueness

## Abstract

Scientists agree that the climate is changing due to human activities, but there is less agreement about the specific consequences and their timeline. Disagreement among climate projections is attributable to the complexity of climate models that differ in their structure, parameters, initial conditions, etc. We examine how different sources of uncertainty affect people’s interpretation of, and reaction to, information about climate change by presenting participants forecasts from multiple experts. Participants viewed three types of sets of sea-level rise projections: (1) precise, but *conflicting*; (2) *imprecise*, but agreeing, and (3) *hybrid* that were both conflicting and imprecise. They estimated the most likely sea-level rise, provided a range of possible values and rated the sets on several features – ambiguity, credibility, completeness, etc. In Study 1, everyone saw the same hybrid set. We found that participants were sensitive to uncertainty between sources, but not to uncertainty about which model was used. The impacts of conflict and imprecision were *combined for estimation tasks* and *compromised for feature ratings*. Estimates were closer to the experts’ original projections, and sets were rated more favorably under imprecision. Estimates were least consistent with (narrower than) the experts in the hybrid condition, but participants rated the conflicting set least favorably. In Study 2, we investigated the hybrid case in more detail by creating several distinct interval sets that combine conflict and imprecision. Two factors drive perceptual differences: *overlap* – the structure of the forecast set (whether intersecting, nested, tangent, or disjoint) – and a*symmetry –* the balance of the set. Estimates were primarily driven by asymmetry, and preferences were primarily driven by overlap. Asymmetric sets were least consistent with the experts: estimated ranges were narrower, and estimates of the most likely value were shifted further below the set mean. Intersecting and nested sets were rated similarly to imprecision, and ratings of disjoint and tangent sets were rated like conflict. Our goal was to determine which underlying factors of information sets drive perceptions of uncertainty in consistent, predictable ways. The two studies lead us to conclude that perceptions of agreement require intersection and balance, and overly precise forecasts lead to greater perceptions of disagreement and a greater likelihood of the public discrediting and misinterpreting information.

## Introduction

Climate forecasts are riddled with uncertainty because climate models involve uncertainties around the model’s structure, the measurement of initial conditions, the parameters of the key variables (e.g., future radiative forcing, population growth, economic activity), and the relationship between these variables. Moreover, because of the interactions between these uncertainties, models are typically run multiple times with different initial conditions and parameterizations, generating a spectrum of predictions to properly capture the deep uncertainties that drive the phenomena. The communication of such deep uncertainty is crucial to allow decision-makers (DMs) to make choices based on an accurate understanding of the state-of-the-art science and strength of the evidence (e.g., Drouet et al., 2015). If scientists do not properly communicate the nature, sources, and magnitude of the uncertainties, the DMs can be either over- or under-confident in the evidence and, in many cases, this can lead to suboptimal decisions (Fischhoff and Davis, 2014). The effects of poorly specified uncertainty can be profound. For example, the North Carolina Sea-Level Rise Assessment Report (N.C. Coastal Resources Commission’s Science Panel on Coastal Hazards, 2010) projected a 39-inch rise in sea-level (ranging from 15 to 55 inches) in the Outer Banks by 2100. In response to this overly precise, long term projection, local conservative groups, worried about the economic devastation associated with this projection, launched an effective campaign against policy initiatives. The local government subsequently banned policy addressing these sea level projections suggesting much valuable real estate would be under water (Siceloff, 2014, News and Observer).

### Climate Model Complexity and Decision-Making

Although there is high agreement among experts about the reality and causes of climate change (CC) (e.g., Doran and Zimmerman, 2009), there is much less agreement among projections of the future climate. Experts disagree on the primary drivers of uncertainty in climate projections including if and how such vital uncertainties can be resolved (e.g., Morgan and Keith, 1995; Zickfeld et al., 2010). For model projections to be useful, stakeholders in areas as diverse as biodiversity, water, transportation, energy, and city and regional planning must resolve the indeterminacy stemming from multiple experts running multiple models with multiple initial conditions producing multiple projections.

When making decisions under deep uncertainty, Decision Makers (DMs) form mental models of the complex systems involved, and these mental models drive subsequent beliefs and behaviors (Newell and Pitman, 2010; Galesic et al., 2016). When mental models are established, even tentatively, DMs evaluate and fit new information into their existing structure and beliefs. Holyoak and Simon (1999) have empirically demonstrated this process for legal decisions: Once an individual reaches a tentative decision, subsequent evaluations of evidence and arguments are affected by the original decision, which in turn influences future decisions. People also distort information to fit their tentatively favored alternative (e.g., Russo and Yong, 2011). Ambiguity in the definition of events, as well as vagueness and imprecision in projected outcomes, allows DMs to interpret results congruently with their own mental models instead of altering their beliefs to incorporate the full range of information (see Kunda, 1990).

### Sources of Uncertainty

The problem of subjective interpretation is magnified when information comes from multiple sources. Research distinguishes between two sources of indeterminacy stemming from multiple sources: conflict and imprecision (Smithson, 1999, 2015). *Imprecision* (sometimes referred to as ambiguity or vagueness) occurs when quantities are specified inexactly and often takes the form of a range of possible outcomes (e.g., “We expect 1–3 inches of snow in the next 24 h”) or an approximation (“We expect about 2 inches of snow”). *Conflict* occurs when quantities cannot simultaneously hold true (“Expert A expects 1 inch of snow in the next 24 h” and “Expert B expects 3 inches”). DMs are generally more conflict averse than imprecision averse (Smithson, 1999), but both conflict and imprecision contribute toward overall perceptions [operationalized by subjective ratings of uncertainty (Smithson, 2015)].

Professionals, such as insurance underwriters and actuaries instinctively differentiate between these sources of indeterminacy. Cabantous et al. (2011) presented insurers with risk estimates for three hazardous events –fires, floods, and hurricanes – from two modeling firms. The models agreed on a mean value (risk), disagreed at either end of a range of values (conflict), or agreed over the same range of values (imprecision). The insurers tended to charge higher premiums for catastrophic risks (e.g., floods) under conflict and higher premiums for non-catastrophic risk (e.g., house fires) under imprecision.

Although imprecision and conflict can operate simultaneously, previous research has focused on the extreme cases where they are distinct. The current studies examine how various sources of uncertainty impact how DMs aggregate, process, and resolve uncertain information from multiple sources in the context of projections related to CC.

### Attitudes Toward Imprecision

Decision-makers generally prefer precise over imprecise options (Ellsberg, 1961; Wallsten et al., 1993; Kramer and Budescu, 2004), but are sensitive to the level of precision and resolution that can be expected in different contexts. As a rule, DMs prefer the most precise option that can be reasonably expected within a specific context. The congruence principle (Wallsten and Budescu, 1995) states that DMs seek congruence between the degree of precision of an event, the nature of the uncertainty surrounding the event, and the representation of the uncertainty. For example, DMs expect very precise estimates of uncertainty for unambiguous events with easily quantifiable uncertainties (e.g., the chance that a man born and residing in the United States will live at least X years). On the other hand, they would probably reject equally precise estimates in the context of ambiguous events with hard to model and quantify uncertainties (e.g., the chance of a *substantial drop* in the national unemployment rate *in the foreseeable future*) and would consider a moderately imprecise estimate to be more credible and informative.

There is also evidence that laypeople do not always prefer precision. Many individuals are imprecision (ambiguity) seeking for unlikely gains and for likely losses (Einhorn and Hogarth, 1985, 1988) for both outcomes and probabilities (Hogarth and Einhorn, 1990; Casey and Scholz, 1991; González-Vallejo et al., 1996; Budescu et al., 2002). For example, a preference for imprecision was demonstrated in financial forecasting by showing that DMs find moderately imprecise financial forecasts to be more credible, more accurate, and induce more confidence than their precise counterparts (Du and Budescu, 2005; Du et al., 2011). Because of the complexity of predicting the future climate, DMs would not expect highly precise predictions (such as a point estimate of the mean global temperature over 50 years). In a task inspired by CC, DMs who could use one of two decision aids, preferred one that graphically showed the full range of values (i.e., stressing and highlighting uncertainty) over one that calculated the expected value of the options and eliminated all uncertainty (Budescu et al., 2014a,b).

### Vagueness as Conflict

Vagueness in complex domains is often driven by expert disagreement. Experts fail to arrive at the same conclusion (whether precise or not) when there are too many unresolved or unknown relationships among variables. The belief that scientists disagree about severity and causes of climate change decreases the endorsement of corrective actions, including policy initiatives, to address the problem. Lewandowsky et al. (2013) show that explaining that scientists agree that humans are causing climate change, increases agreement that climate change and certain climate trends (increased temperature, sea-level, and natural disasters) are attributable to human activity. Differences in perceptions of a scientific consensus are driven by individuals’ worldview, measured both by the strength of their belief in the free-market (Lewandowsky et al., 2013) and their cultural cognition – a theory describing how the values associated with cultural identity determine beliefs (Kahan et al., 2011).

Decision-makers must decide how to weight competing experts’ forecasts based on their information, knowledge, ability, beliefs, etc. Disagreement among experts can be attributed to features of the experts, such as competence, knowledge, bias, or their candidness about uncertainty and to environmental factors, such as complexity and stochasticity (Hammond, 1996; Shanteau, 2000; Dieckmann et al., 2017). Interestingly, the public’s knowledge and ability drive their perceptions of the experts’ knowledge and ability, so that DMs with less topic knowledge and who are less numerate are more likely to attribute expert disagreement to incompetence (Dieckmann et al., 2015). More knowledgeable DMs attribute the conflict to various biases and conflicts of interest, while more numerate DMs attribute it to the stochastic nature of the events. DMs must reconcile disagreeing forecasts by aggregating the available information with their own beliefs. When individual judges combine forecasts, they are sensitive to the structure of the information and the nature of their cognitive processes (Wallsten et al., 1997). Simple aggregation methods, like averaging, are often highly accurate and robust (Clemen, 1989). However, DMs often fail to understand the benefits of averaging for reducing individual error (Larrick and Soll, 2006), and fail to adjust their own beliefs sufficiently to incorporate the advice of others (Yaniv and Milyavsky, 2007).

### Communication of Vague Information

The presentation of uncertain information is a tradeoff between providing enough precision to be useful while being sufficiently imprecise to be realistic. The communicator and audience often have competing goals. Communicators prefer to communicate vaguely, and audiences prefer precise information (e.g., Erev and Cohen, 1990; Wallsten et al., 1993). The description of uncertainty around climate change has ultimately led to a divide between public and scientific perceptions of the problem (Zehr, 2016), even though greater uncertainty leads to a greater expectation of risk and damage (Lewandowsky et al., 2014).

Risk communication experts recommend transparency about uncertainty to aid interpretability for DMs (Fischhoff and Davis, 2014). A simple and common communication tool is to provide range estimates, such as confidence intervals, to express the scope of values considered reasonably possible or probable. Uncertainty about climate change, when presented as a range estimate, is considered more credible when certainty is not possible (MacInnis et al., unpublished). Interval estimates are perceived to be more credible in hindsight and to have higher utility for deciding at higher likelihoods (Dieckmann et al., 2010). Vagueness that characterizes both numerical ranges and verbal ambiguity, interacts with message framing resulting in vagueness avoidance for positively framed values and vagueness seeking for negatively framed values (Kuhn, 1997). Ranges can improve attributions of the likelihoods across possible outcomes (Dieckmann et al., 2012) and can improve the appropriateness of steps taken to address weather-related risks (Joslyn and LeClerc, 2012).

### The Current Paper

We examine DMs’ reactions to sources of uncertainty arising from multiple forecasts in the context of CC. Climate is a perfect domain for such studies. Due to the computational constraints of running complex climate models, it is often impossible to resolve these disagreements and indeterminacies during modeling. There is a tradeoff between model resolution and expected accuracy in climate models. Modelers can set model parameters to estimate the future climate with high resolution (say at the level of a county or a neighborhood), but higher resolution can reduce confidence in the accuracy of their forecasts. Conversely, lower resolution forecasts (e.g., global models) may be perceived as more accurate but uninformative or even irrelevant at a local level. This tradeoff is understood by laypeople who intuitively rate narrower intervals as more informative than their precise counterparts (e.g., Du et al., 2011), but less likely to be accurate, and vice versa (Yaniv and Foster, 1995). Therefore, to maintain a high degree of confidence, climate projections are typically expressed with equivocacy by including vagueness or uncertainty information.

In two studies, we present respondents with two forecasts related to future impacts of CC with various sources of indeterminacy and disagreement. Consistent with the CC literature we differentiate between *model and source uncertainty*. Model uncertainty describes indeterminacy stemming from the models’ structure and inputs, and source uncertainty describes disagreement between how expert sources utilize models and interpret their output. The forecasts – which are projections of sea level rise and their impact on the ports in southern California over the next 50 years – vary in whether they came from the same or different models and use the same or different initial conditions. In addition, the various sets of experts’ forecasts reflect different degrees of imprecision and conflict. In each case, the respondents estimated the most likely value as well as a range of possible values. Additionally, we obtained their confidence in those estimates in two ways: direct ratings and willingness-to-pay for insurance to reduce the risk of losing money when betting on their estimates. Finally, they provided comparative ratings of the forecast sets on key attributes.

## Study 1

We test how DMs react to various sources and types of uncertainty underlying model projections. More specifically, we test a 3 × 4 typology of uncertainty. One factor consists of three levels of *source uncertainty* that describe how forecasts from two experts relate to each other – conflict between precise experts, imprecise agreeing experts, and a hybrid case which includes both conflict and imprecision. The second factor – *model uncertainty* – is based on a 2 × 2 crossing of *structural uncertainty* about the model and uncertainty reflecting *judgmental* interpretation of the models’ results. We manipulate structural uncertainty by providing projections from the same, or different, models, and we manipulate judgmental uncertainty by providing projections from one or two sets of initial conditions. See **Table 1** for a schematic description of the design.

**Table 1:** Typology of source and model uncertainty.

Model uncertainty (Between subjects)		Source of uncertainty (Randomly ordered within subjects)		
	
Structural uncertainty	Judgmental uncertainty	*Conflict* between two precise forecasts	Two identical *Imprecise* forecasts	*Hybrid*: Two conflicting imprecise forecasts
One model	One set of initial conditions			
	Two sets of initial conditions			
Two Models	One set of initial conditions			
	Two sets of initial conditions			


We test the effects of these various facets of uncertainty on three distinct sets of dependent variables. The first two sets – estimates of the target quantity and its feasible bounds, and confidence in the estimates – are obtained for each case. The third set consists of a group of concurrent, comparative ratings of the various cases obtained after making all estimates. This set provides a retrospective global evaluation of the various sources of uncertainty.

Overall, our goal is to test how sensitive the DMs’ estimates and expressions of confidence will be to the manipulations of the various facets of uncertainty. Consider first the effects of the source of uncertainty. We do not expect any differences in terms of the best estimates of the target quantity which will, invariably, be some simple aggregate of, or compromise between, the two extreme – the lowest and the highest – values presented by the forecasters^1^.

We have differential expectations about the range of the estimates and the confidence they inspire. We expect that the DMs will be most faithful to (deviate least from) the experts’ estimates in the case of identical and imprecise forecasts, and we expect that the range of estimates will be the narrowest in the hybrid case that, by its nature, highlights a narrow area of agreement, so we expect the DMs to focus on it. In line with previous results (Smithson, 1999; Baillon et al., 2012), we expect the lowest levels of confidence and the highest willingness to purchase insurance in the presence of conflict between the experts. Similarly, we expect that, in retrospect, DMs will rate the conflicting cases lowest (least desirable or attractive) in all respects, and rate the imprecise cases highest on most attributes, with the hybrid cases in between.

We expect that DMs will be less sensitive to our manipulations of model uncertainty which are more subtle and, unlike source uncertainty which is very direct and salient, reflect a deeper understanding and analysis of the situation. In general, we expect that lower (structural and judgmental) uncertainty will induce narrower ranges of estimates, higher levels of confidence and less willingness to purchase insurance. Finally, we predict that DMs will react more strongly to information that reduces *structural* (rather than *judgmental*) uncertainty, because structural uncertainty implies there is conflict in the relationship between physical phenomena while judgmental uncertainty, can be attributed to changes in initial conditions or model parameters, and can be resolved more easily.

### Methods

#### Participants and Design

One hundred and thirty undergraduate and graduate students from New York University and Fordham University participated in the study. Eighty-seven (67%) were female, and the mean age was 20.9 (*SD* = 2.8). Participants received $10 for participating and a performance-based prize (up to an additional $20; described below). We varied model uncertainty – a combination of two binary factors reflecting structural and judgmental uncertainties – between subjects. Participants were randomly assigned to one of four groups, and they saw projections from either one or two model(s), reflecting structural uncertainty, and using one or two set(s) of initial conditions, capturing judgmental uncertainty. We varied source uncertainty within-subjects, so all participants saw three projection sets from various pairs of experts: one set of precise, but conflicting projections; one set of imprecise, but agreeing, projections; and one set of *hybrid* projections that were both conflicting and imprecise (see **Table 1**).

#### Material and Procedure

Participants saw the same scenario – describing the effects of sea level rise on ports in Southern California – each time with a different set of projections (see Supplementary Materials for the full scenario). Additional tasks interspersed between them served as distractors from the manipulation. Each projection set consisted of two forecasts from two different experts. In each condition, the pairs of forecasts were attributed to the same two experts. These experts were labeled generically, so no specific valence or ideology could be ascribed to either expert. The three source uncertainty conditions were presented in random order; all three versions had the same mean (25 inches) and the same range (10–40 inches) of sea level rise projected over 50 years. The conflict condition consisted of disagreeing point estimates (10 vs. 40 inches), the imprecision condition consisted of agreeing interval estimates (both 10–40 inches), and the hybrid condition consisted of two non-overlapping intervals with a common endpoint (10–25 inches and 25–40 inches). To highlight why similar models could result in different output, the following statement was added to each scenario, “Note: Differences between projections may reflect the experts’ uncertainty about the values of the key parameters.”

**FIGURE 1:**
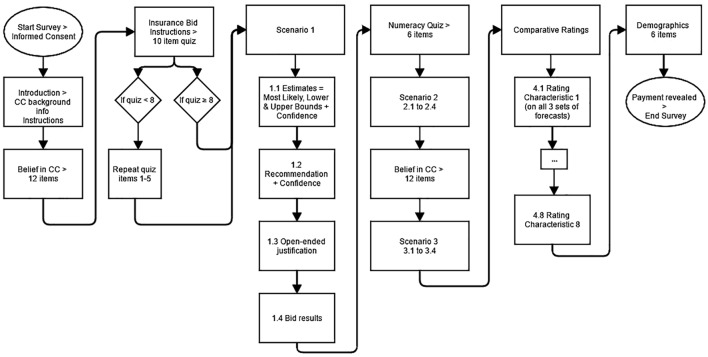
Study flow for one participant.

**Figure 1** presents the flow of the study from beginning to end. Participants started by reading background information about climate models including the basic science behind CC its potential impacts, a description of Earth System Models (EaSMs), and an explanation of why they are uncertain (see Supplementary Materials for introductory text). We developed this text in consultation with climate scientists, ecologists, and water experts. This information provided a basic understanding of the sources of imprecision and disagreement among the experts and model projections, even when they agree on the general science of CC.

Next, participants completed a 12-item belief in CC inventory. All items were five-option Likert statements (labeled from “strongly disagree” to “strongly agree”) that were adapted from Heath and Gifford (2006). There were six subscales of perceptions of CC including general belief in, personal experience with, belief in humans causing, belief in serious consequences of, self-efficacy to make a difference in, and intentions to take alleviative actions to address, climate change. The original subscales include 4 items each, but we administered only 2 of the items to shorten the experiment time. We conducted a factor analysis on 374 responses (from the US sample of Budescu et al., 2014b) using a single factor and retained the top two items (i.e., with the highest loading) in each. The reduced scale maintained the level of reliability of the full scale and maintained or improved upon the reliability for all subscales except one (personal experience dropped from α = 0.87 to 0.80; see Supplementary Table S1 in the Supplementary Material for the reduced scales). Participants were credited with an endowment of $10 for completing the 12 belief items for use in the first (of three) incentivized betting task to determine their underlying uncertainty.

Participants then read a one paragraph summary of a scenario regarding the need to raise the ports in Southern California to protect against projected sea level rise. Participants saw two expert projections pertaining to the scenario. The experts were labeled generically (e.g., Scientist A and B) and the models were given fictitious names Global Circulation Simulation model - version X (GCSX) and Earth System Generation model - version Y (ESGY).

After reading the scenario, the participants provided their best guess of the target value, stated a range of likely values, and rated their confidence on a seven-option Likert scale from “not at all” to “extremely.” They then were asked to imagine they were a consultant to the port authority and recommend a value to plan for and rate their confidence on the same seven-point scale. They were also allowed to give an open-ended justification for their estimates, but an analysis of the content is beyond the scope of this paper.

To motivate the subjects to provide honest estimates, they were told that their estimates for a scenario will be part of a bet that potentially paid based on its accuracy. If their estimate was within 10% of the “true” value, they could double their $10 endowment, and if their estimate was off by more than 10%, they would lose their endowment. The true values were generated by calculating the mean of ten runs assuming expert projections was distributed normally over the range. Before resolving their bet, participants were offered the opportunity to purchase insurance in a bidding procedure adapted from Becker et al. (1964), by using the $10 they earned previously. Specifically, they could bid any amount $B ($0 ≤ B ≤ $7.50) to purchase insurance. If their bid was at least as large as our randomly generated counteroffer (between $0 and $5, in increments of $.25), they were insured, and their loss would be reduced to $2.50 plus the cost of insurance (which was equal to the counteroffer). If their bid was less than the counteroffer, they were uninsured. This procedure was designed to elicit bids that accurately reflect the DM’s perceived uncertainty.

Participants were told that overbidding can lead to overspending on insurance and underbidding can lead to under-protection, and they completed a quiz about the bidding procedure. The quiz provided an example including a best guess, insurance bid, counteroffer, and true value. Participants were asked if the bid was successful in purchasing insurance, the price of insurance, if the bet was successful, winnings/losings, and total payment for the example. Participants answered these five questions for two different examples. If they answered fewer than 8 questions correctly, they repeated the first example and the associated quiz questions.

After completing all the stages of the first scenario, the participants took a six-item numeracy quiz which served as a distractor and to control for numeracy since it has been found to be a strong predictor of decision-making skill (e.g., Cokely et al., 2018). We adapted our numeracy quiz from the eight-item Weller et al. (2013) scale dropping two items, for being too time consuming. Participants were credited with a $10 endowment to use in the second bet for completing the numeracy quiz.

After completing all stages – estimates and insurance bids – of the second scenario, participants completed the same 12-item belief in CC inventory to test the reliability of this measure and as a distractor before the third and final projection set. They were credited with their third (and final) $10 endowment after the completion of this inventory and completed all stages – estimates and insurance bids – of the third scenario.

The estimation and insurance bid on the second and third procedure were identical to the original one. The labels identifying the experts and models and number of initial conditions were fixed across scenarios within participants. Only the values and structure of the forecasts varied across scenarios.

After completing the third projection set, participants concurrently rated the three projection sets on eight attributes: ambiguous, conflicting, precise, credible, likely to be accurate, informative, complete, and easy to reconcile/decide. All three projection sets were shown on the screen and subjects were asked to rate all three projection sets independently for each attribute using a 7-point Likert scale from “not at all” to “extremely.” Each trait was presented on a separate screen in a random order.

Finally, the participants answered some basic demographic questions including age, sex, major of study, year in school, political affiliation (Republican, Independent, Democrat, or other), and strength of political identity (five-options from “very weak” to “very strong”), and they received the winnings of one randomly selected bet.

### Results

We ran a series of 3 × 2 × 2 mixed MANCOVAs with source uncertainty as the within-subjects factor and (number of) models and initial conditions as between-subjects factors and numeracy as a covariate^2^. Several results stand out: (1) We did not find any significant differences across conditions for the participants’ best estimates and their recommendations, as expected, so we report their personal estimates throughout the results; (2) We did not find significant effects of the two components of model uncertainty (structural and judgmental) on any dependent variable; (3) We found several systematic effects of source uncertainty which we describe and discuss below one dependent variable at a time (**Table 2** shows the means and standard errors of all estimates by source uncertainty); (4) We did not find order effects: response patterns do not change if we only analyze the first condition seen by each participant, so differences cannot be attributed to the influence of previous judgments.

**Table 2:** Descriptive statistics of estimates and confidence by source uncertainty.

DV	Overall			Imprecision			Conflict			Hybrid		
				
	*N*	Mean	*SE*	*N*	Mean	*SE*	*N*	Mean	*SE*	*N*	Mean	*SE*
Best guess	390	25.40	0.22	130	25.88	0.33	130	25.10	0.42	130	25.21	0.36
Lower bound	388	13.71	0.26	129	13.22	0.43	130	13.62	0.50	129	14.29	0.42
Upper bound	382	35.59	0.30	128	36.55	0.47	128	35.38	0.56	126	34.81	0.52
Range	380	21.69	0.46	127	23.28	0.75	128	21.67	0.84	125	20.09	0.75
Confidence	390	4.02	0.07	130	4.28	0.14	130	3.78	0.12	130	3.98	0.12


#### Range Estimates Across Sources of Uncertainty

**FIGURE 2:**
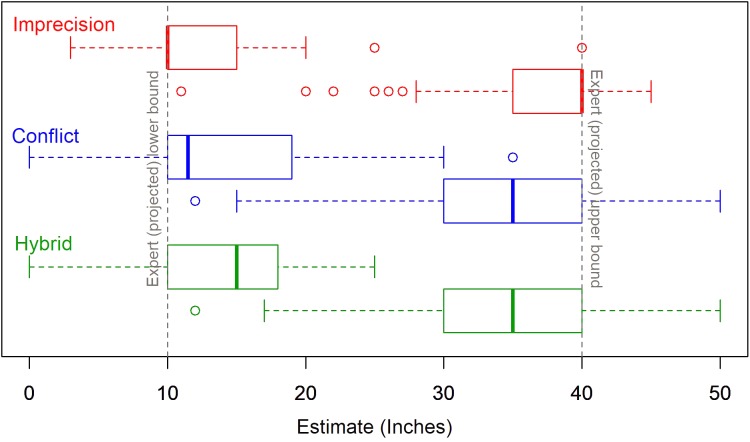
Bound estimates by source uncertainty (Study 1).

The lower bound, upper bound, and range estimates all varied significantly by source uncertainty. In all the conditions, the two estimated bounds were shifted away from the expert projections toward the center of the intervals. In other words, the judged lower bounds are higher than the lower forecasts, the judged upper bounds are lower than the upper forecasts and, consequently, the judged ranges are narrower than the actual range of forecasts. The estimates vary significantly across conditions: *F*_(2,123)_ = 4.09, *F*_(2,120)_ = 9.37, and *F*_(2,119)_ = 12.43 for the lower bounds, upper bounds and the range, respectively. **Figure 2** shows boxplots of the bound estimates by source uncertainty. The DMs’ range estimates are most consistent with the forecasts in the imprecise condition; the median bounds under imprecision are, essentially, equal to the experts’ forecasts. On the other hand, we observed the greatest reduction in range in the hybrid condition – the median range for hybrid sources is reduced by about one third – suggesting that the distinct effects of imprecision and conflict are cumulative.

### Confidence and Insurance Across Sources of Uncertainty

There was a markedly different pattern of confidence and willingness-to-pay for insurance as a function of source uncertainty *F*_(2,125)_ = 9.01 and 6.51 for estimates and recommendations, respectively. DMs were most confident under imprecision and least confident under conflict. Conversely, the mean insurance bids were lowest for imprecision and highest under conflict. The two patterns were consistent, since a higher insurance bid implies lower confidence. DMs used different bidding strategies under imprecision compared to conflict and the hybrid source of uncertainty. Recall that the minimal possible bid was $0 and the maximal was $7.50. We considered all small bids (≤$0.25) as a rejection of insurance, very high bids (≥$7.25) as a commitment to purchasing insurance, and moderate bids ∈ ($0.25, 7.25) as reflecting a more nuanced conditional approach (purchase “if the price is right”). Most DMs (87%) followed the same strategy for all three sources of uncertainty, and we found no difference in the proportion of DMs who placed conditional bids (between 84% and 85%) in all conditions. DMs were more likely to reject insurance than purchase it under imprecision (12% vs. 5%). Conversely, they were more likely to commit to purchasing insurance under conflict (9% vs. 6%). In the hybrid condition the two rates were similar (8% vs. 6%). The difference between conflict and imprecision was significant, χ^2^_(2)_ = 8.27, *p* = 0.02 using Stuart–Maxwell test for matched categories, with 11% of DMs more likely to purchase insurance under conflict, and only 2% more likely to purchase insurance under imprecision.

### Retrospective Ratings Across Sources of Uncertainty

**FIGURE 3:**
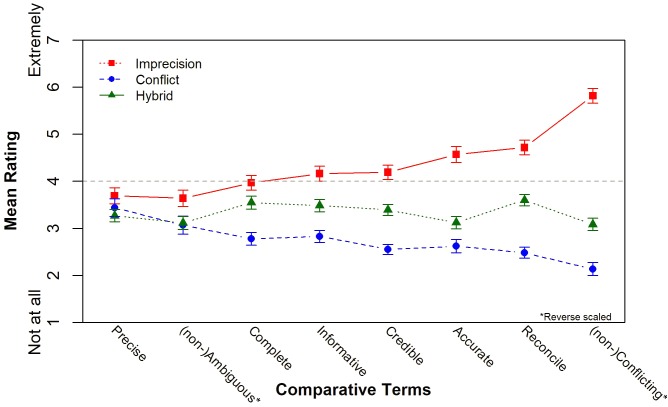
Comparative rating means by source uncertainty (Study 1). Error bars reflect standard error of the mean for each condition.

We reverse scored “ambiguous” and “conflicting,” so that high values refer to positive valence for all attributes. **Figure 3** shows the mean ratings by source uncertainty. We found no differences in the ratings of “precision,” but for the other seven attributes the differences were significant: On average, DMs rated the imprecise set highest and the conflicting set lowest. The largest difference between the three sources was observed for the rating of (non-)conflicting scale, and the smallest difference was observed for the (un-)ambiguous scale. The hybrid set was consistently rated in between the other two sets for all seven significant attributes, but its distance from the two extreme conditions varies as a function of the attribute. Mean ratings of the hybrid sets more closely resemble the mean responses under conflict for “(non-)conflicting,” “(un)ambiguous,” and “easy to reconcile/decide.” On the other hand, the mean hybrid ratings were more like the mean ratings of the imprecise set for “completeness.” The mean hybrid ratings were equidistant from imprecision and conflict for the other three attributes: “informative,” “credible,” and “(likely to be) accurate.”

## Study 2

In the second study, we focus on the intriguing, and previously unstudied, hybrid cases by examining the differential impact of various patterns of (interval) projections sets obtained from pairs of forecasters. The design is similar to Study 1, with some minor changes: Each DMs saw one of 30 different hybrid projection sets – involving distinct combinations of conflict and imprecision – in addition to the same set of conflicting (and precise) forecasts and the same imprecise (and agreeing) set. We also simplified the willingness-to-pay for insurance task. Rather than bidding for insurance with the BDM procedure, DMs could choose to purchase one of four levels of insurance at different pre-determined prices. Considering the results of Study 1, we did not manipulate model uncertainty. Thus, we only study source uncertainty and our hypotheses are focused on the hybrid projection sets.

We expect that DMs will react more strongly to information that reduces perceived conflict than perceived imprecision because DMs tend to be more conflict averse than imprecision averse (Smithson, 1999), as we confirmed in Study1. Following Study 1, we expect that both conflict and imprecision will contribute toward overall uncertainty, and when combined (as a hybrid or mixed source condition) their effects will be aggregated differently based on the task: (a) DMs will use a weighted mean for global preferential judgments including confidence, so we expect contributions toward overall uncertainty in the following pattern: ambiguity < hybrid < conflict, but (b) they will have combined effects for estimation tasks, so we will observe the following pattern of shifting away from the experts: ambiguity < conflict < hybrid.

We develop a systematic typology of hybrid patterns and predict that the DMs’ responses will vary based on the two key factors of this classification – overlap and (a)symmetry. We expect that the type and degree of overlap between the two estimates will have a stronger influence on the global ratings than the level and nature of asymmetry between the estimates, because overlaps will drive the perceived agreement between projections. On the other hand, the degree of (a)symmetry should have a stronger influence on quantitative estimates than overlap because a large degree of asymmetry signals that averaging may not be the best method of aggregation compared to other methods (like using the median).

### Methods

#### Participants and Design

A total of 1,084 participants completed the study online. They were recruited both via Fordham University’s business school subject pool (12%) and via a Qualtrics national panel (88%). The former group received course credit, and the latter received Qualtrics’ standard honorarium for completing the study. Since there were no differences between the two groups of subjects we combine them in the analyses. In addition, 10% of all participants were randomly selected to receive an additional cash incentive of up to $14 based on their performance on one randomly selected task.

Responses were pre-screened for validity by the following pre-determined criteria to remove responses with inadequate effort: Participants must have (1) completed the survey, (2) taken at least 6 min to complete it, (3) had fewer than 15% of responses missing, and (4) straight-lined (answered identically) on at most 10 (out of 14) pages. Fewer than 14% of the responses did not meet the minimum criteria (the valid response rate did not vary by recruitment method), so we analyze a total of 937 valid responses. The sample was 50% female, the mean age was 44.4 (*SD* = 15.7). About 32% each self-identify as Democrats and Independents, and 28% as Republicans. Most respondents (45%) had at least a college degree, 33% had some college credit, and 22% had no college education.

Given the clear results of Study 1, we did not manipulate model uncertainty, so all participants saw forecasts from two experts and two models for each set of forecasts. The source of uncertainty was varied within-subjects, and participants saw one set of conflicting, and precise forecasts, one set of imprecise, agreeing, forecasts and one hybrid set. The unique feature of this study is that participants were randomly assigned to one of 30 conditions differentiated by 30 distinct hybrid conditions.

The various projection sets can be characterized by the type and degree of their *overlap* and (a)*symmetry*. We distinguish between four categories of overlap: (1) *intersecting* sets are partially overlapping, (2) *nested* sets feature one interval as a subset of (embedded within) the other, (3) *tangent* sets include intervals that share a common endpoint (as in Study 1), and (4) *disjoint* sets do not overlap. Let *LB1* and *LB2* be the lower bounds of the two intervals and let *UB1* and *UB2* be their corresponding upper bounds. Without loss of generality, we assume that *LB1* ≤*LB2* and *UB1* ≤*UB2* so, in other words, the first interval is lower and the second is higher. We define a measure of Degree of Overlap, *DO*, that measures the size of the interval of each set that is (dis)similar (i.e., intersecting, nested, or disjoint).

$$\text{Degree of Overlap=DO=}\left\{\begin{array}{c} UB1-LB2\,\;if\,\;non-nested\\ UB2-LB2\,\;if\,\;nested \end{array}\right\}$$

**FIGURE 4:**
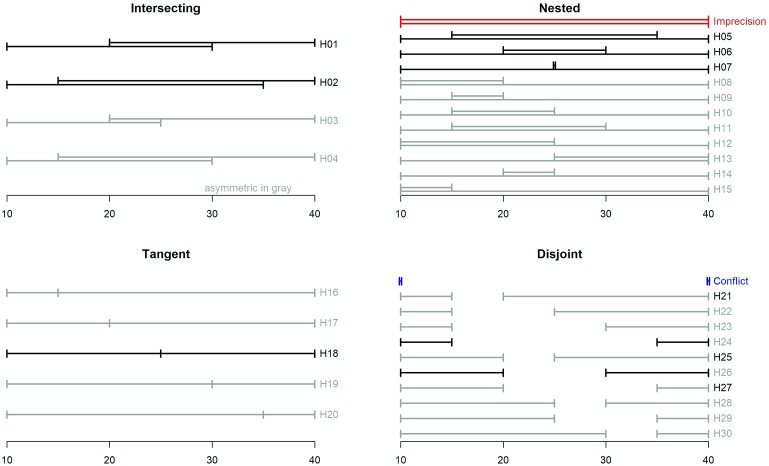
Graphical representation of the pairs of interval forecasts used as stimuli for Study 2.

*DO* is positive for intersecting and nested sets, negative for disjoint sets, and 0 for tangent sets. For example, the interesting set H01 and the nested set H06 have equal DOs, while the disjoint set H26 has a negative DO of equal magnitude (see **Figure 4**).

There are many ways to define the degree of (a)symmetry of the two sets. The basic definition we use to describe the design is based on the distance between the midpoints of the two intervals from the midpoint of all estimates. Let *M* be the midpoint of all four points, and *m*_i_ be the midpoint of the *i*’th interval (*i* = 1,2). Formally: $M=\frac{\max(UB)-\min\left(LB\right)}{2}$ and $m_{i}=\frac{UBi-LBi}{2}\left(i=1,2\right)$. We define:

$$Asymmetry=AS=|M-m_{1}|-|M-m_{2}|.$$

If the midpoints of both intervals are equidistant from *M*, they are considered symmetric (*AS* = 0). If the midpoint of the lower (upper) interval is farther from the center, *M*, then the set is positively (negatively) skewed. For example, set H16 has a positive skew, and set H20 has a negative skew of equal magnitude. All forecast sets cover the same 30-inch range (from 10 to 40), so DO and AS for each set can be thought of as the breadth of that range that is overlapping (or not) and unbalanced.

#### Materials and Procedure

Participants were randomly assigned to one of 30 different hybrid conditions. Each included a set consisting of two interval projections that are both conflicting and imprecise to various degrees. These sets were constructed by varying the type and degree of overlap (intersecting, nesting, tangent, or disjoint) and asymmetry (symmetric or skewed), so they span the full 4 × 2 typology of the two factors. The complete classification is displayed in **Figure 4**.

We used the same procedure as in Study 1 with some minor modifications. Participants started by reading the informed consent and a shortened version of the background information. We used the same scenario describing how sea-level rise will affect California ports (see Supplementary Materials) showing two accompanying forecasts, but we fixed the number of experts (2) and the number of models (2). The projections were presented in a chart, and we randomized the order of presentation for each pair, so either the higher or lower projection could appear on the left or right. Participants performed the same estimation tasks and confidence ratings, but they did not provide recommendations as a government consultant. Instead, we asked them to provide a 90% probability interval, so we had a benchmark to compare the width of their range estimates.

We altered the willingness-to-pay for insurance item because the bidding procedure was time-consuming, and some participants struggled to understand it. After making their estimates, participants chose one of four levels of insurance with different levels of coverage and, of course, different costs. They could choose to (1) be uninsured, (2) pay $1 to reduce their possible loss from $7 to $5, (3) pay $2 to reduce their possible loss to $3, or (4) pay $3 to reduce their possible loss to $1^3^.

We also shortened the belief in CC inventory to two lists of 10 items each. In total there were two items from each subscale one of which was repeated in both lists. Beyond the six repeated items, there was one item for each subscale that was not repeated, so participants saw half of the non-duplicated items in each list. We added an item calling for self-assessment of knowledge in CC (using the same five-option scale). We used a four-item numeracy test including two items from Schwartz et al. (1997) and two items from the cognitive reflection test (Frederick, 2005) (see Supplementary Materials for scale items).

In the comparative questionnaire we dropped the rating of the trait “informative,” because of its high similarity to “complete,” and in the demographic questionnaire we replaced the major and year in school with highest level of education because most of the Qualtrics respondents were not students.

### Results

We ran 4 × 2 × 3 mixed MANOVAs with source uncertainty (imprecision, conflict, or hybrid) as the within-subjects factor. The 30 hybrid cases were combined into four types of overlap (intersecting, nested, tangent, or disjoint) and two levels of symmetry (symmetric vs. asymmetric) and defined the between-subjects factors. We report first the results pertaining to the source of uncertainty, which replicate Study 1.

#### Estimates as a Function of Source of Uncertainty

We replicated the key results regarding the range estimate from Study 1. The lower and upper bounds, ranges, and the confidence ratings (see **Table 3**) varied significantly across the sources of uncertainty. DMs estimated the widest ranges under imprecision and the narrowest in the hybrid conditions. The best estimates systematically underestimated the mean of the experts’ forecasts, and the deviation from the mean of the experts’ forecasts was significantly greater when the two experts disagreed (conflict).

**Table 3:** Means by source uncertainty.

Outcome	Source					
	
	Imprecision		Conflict		Hybrid	
			
	Mean	*SE*	Mean	*SE*	Mean	*SE*
Estimate (shift)	-2.18	0.28	-3.66	0.29	-2.17	0.29
Lower bound	13.56	0.24	12.67	0.24	14.07	0.25
Upper bound	31.33	0.37	29.64	0.39	29.89	0.38
Range	17.77	0.37	16.97	0.35	15.81	0.33
Confidence (est)	4.39	0.05	4.23	0.05	4.23	0.05
Insurance bid	1.19	0.04	1.24	0.04	1.23	0.04
Confidence Interval	17.47	0.44	16.99	0.44	16.11	0.40


#### Confidence and Ratings as a Function of Source of Uncertainty

The analysis of the mean confidence and attribute ratings replicated the patterns from Study 1. DMs were most confident under imprecision, but there were no differences between conflict and the hybrid sets, and there were no significant differences across various levels of overlap or (a)symmetry. The pattern of insurance bids was similar to Study 1 with participants most likely to decline, and least likely to purchase the highest level of insurance, under imprecision. However, that difference was not significant, and fell short of a small effect, χ^2^_(6)_ = 5.64, *p* = 0.46, Cramer’s *V* = 0.03.

**FIGURE 5:**
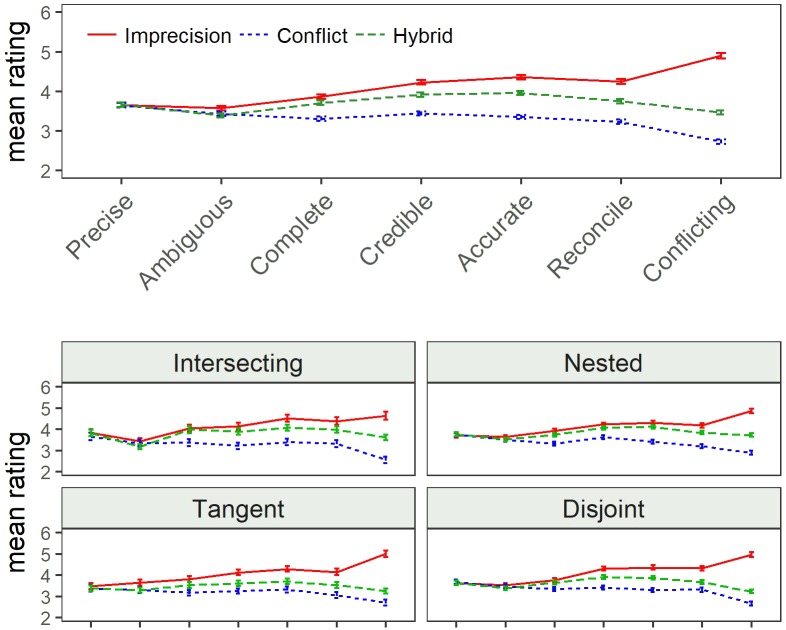
Comparative ratings by structure of overlap.

Replicating the pattern from Study 1, the imprecise set was rated highest and the conflicting set was rated lowest for most attributes (there are no difference in the “precision” ratings). It is striking how stable the ratings were in their preference for imprecision and aversion to conflict for all four structures. There were main effects of source uncertainty on the ratings for the structure of overlap, but not differences between levels of asymmetry. The mean ratings of intersecting and nested hybrid sets were similar to ratings of the imprecise set and the mean ratings of the disjoint and tangent hybrid sets were similar to ratings of conflict. There were significant differences for the ratings of *ambiguous* and *conflicting*: nested and disjoint sets were rated the most ambiguous, and tangent and then intersecting were the least. **Figure 5** displays the mean ratings for all attributes by source of uncertainty and structure of overlap. Participants were most likely to decline insurance when viewing tangent and disjoint groups, and least likely when viewing nested and intersecting, but the effect was small and not significant, χ^2^_(9)_ = 9.29, *p* = 0.41, Cramer’s *V* = 0.03.

The unique and novel feature of this study is the use of 30 hybrid conditions that vary along several attributes which allow us to analyze and determine if, and why, DMs are sensitive to imprecise and conflicting forecasts. Next, we discuss some of these results separately for the various dependent variables. To capture and model the subtle effects associated with various degrees of overlap and asymmetry of the 30 hybrid cases, we use regression models using the degree of each major factor and their interaction as predictors. We conducted separate regressions to predict the mean and variance of each estimate of the 30 hybrid sets allowing us to test how the key factors affect the magnitude as well as variability of the estimates. Most DMs, as expected, gave estimates close to the mean and the bounds of the set, and a small – but non-trivial – number of DMs gave greatly different responses. Focusing on the mean and variance of each group allows us to focus on the typical respondent and minimize the influence of unusual individuals.

#### Estimates as a Function of (A)Symmetry of the Sets

**FIGURE 6:**
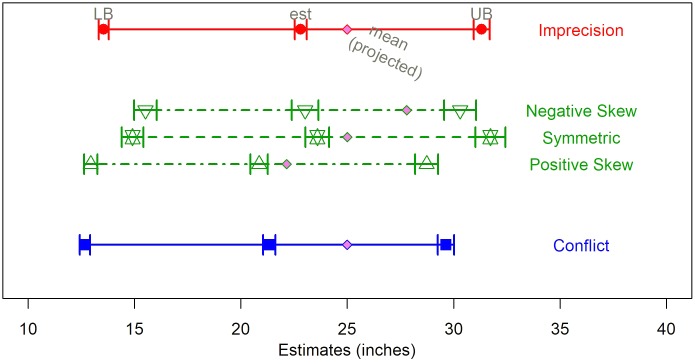
Estimates (best, lower, and upper bounds) by level of asymmetry. Each source has a unique color, and each condition has a unique symbol. Responses to the Hybrid sets are averaged across direction of skewness. Error bars display the standard error of the mean for each condition.

We ran regression models predicting each of the DMs’ estimate using the degree of overlap, degree of asymmetry, and their interaction as predictors. The degree of asymmetry was a significant predictor of the means of all three estimates – most likely value, lower bound, and upper bound – but not for the estimated ranges, the subjective 90% probability intervals, or the confidence ratings (see **Table 4**). **Figure 6** displays the set of mean estimates by level of asymmetry. Generally, there was more shifting away from the experts’ upper bound than the lower bound, and the best estimates were consistently shifted below the set mean, suggesting that DMs act as if the forecasts overestimate the “true” value. As expected, the positively skewed sets were shifted considerably toward the lower end. The symmetric set was closer to the center, but slightly shifted to the lower end. Estimates for the negatively skewed sets curbed expectations. The estimated mean was closer to the midpoint than the set mean, and the upper bounds were greatly shifted in the negative direction.

**Table 4:** Regression models of mean and variance of estimates by overlap and asymmetry.

Outcome	Source	Best estimate				Lower bound				Upper bound			
				
		*B*	*SE*	DW		*B*	*SE*	DW		*B*	*SE*	DW	
Mean	Intercept	-2.58	0.33			14.46	0.28			30.20	0.41		
	Overlap	0.02	0.03	0.01		-0.01	0.03	0.01		0.02	0.04	0.00	
	Asymmetry	0.27	0.06	0.22	^∗^	-0.23	0.05	0.24	^∗^	-0.22	0.08	0.13	^∗^
	O’lap × Asym	0.00	0.01	0.22		0.00	0.01	0.24		0.00	0.01	0.13	
	Adj-*R*^2^			0.39				0.43				0.17	
Variance	Intercept	81.62	6.49			58.28	6.67			131.81	11.18		
	Overlap	-0.86	0.59	0.04		-1.30	0.61	0.06	^∗^	-0.30	1.02	0.00	
	Asymmetry	-1.53	1.24	0.02		-2.26	1.27	0.04		0.01	2.13	0.00	
	O’lap × Asym	0.13	0.16	0.08		0.23	0.16	0.15		0.03	0.27	0.00	
	Adj-*R*^2^			0.03				0.16				-0.11	


**^∗^Significant at p < 0.05; DW, general dominance weight.**

Most likely estimates were shifted further below the set mean for negatively skewed sets (-4.78, *SE* = 0.62) than both symmetric (-1.41, *SE* = 0.55; mean diff. = 3.37, 95% C.I. = [1.45, 5.29], *p* < 0.001) and positively skewed sets (-1.29, *SE* = 0.41; mean diff. = 2.76, 95% C.I. = [0.80, 4.73], *p* = 0.003)^4^. There was significantly less shifting away from the lower bound when the set had a positive skew (12.94 (*SE* = 0.31) compared to 14.91 (*SE* = 0.51), mean diff. = 1.97, 95% C.I. = [0.59, 3.35], *p* = 0.002 for symmetric and 15.51 (*SE* = 0.54), 2.58, 95% C.I. = [1.17, 3.99], *p* < 0.001 for negatively skewed sets), and significantly lower upper bound estimates for positively skewed sets (28.73, *SE* = 0.55) compared to symmetric sets (31.73 (*SE* = 0.70), mean diff. = 3.00, 95% C.I. = [0.86, 5.13], *p* < 0.003), but no difference from the negatively skewed sets (30.31, *SE* = 0.75).

#### Estimates as a Function of Overlap

**FIGURE 7:**
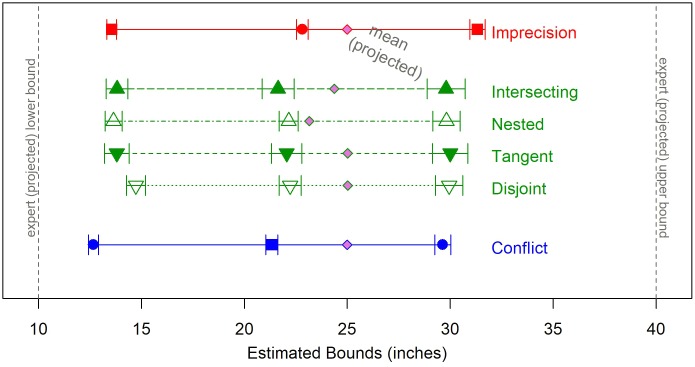
Estimates (best, lower, and upper Bounds) by category of overlap. Each source has a unique color, and each condition has a unique symbol. Responses to the Hybrid sets are averaged across structure of overlap. Error bars display the standard error of the mean for each condition.

The differences between the four overlap categories (intersecting, nested, tangent, or disjoint) are less pronounced. **Figure 7** displays their respective mean estimates. There was a significant difference in shift from the set mean between the categories of overlap [*F*(3,926) = 3.03, *p* = 0.03], which was driven by the difference between disjoint sets, which show the largest shift, and nested sets, with the smallest (mean diff. = 1.79, 95% C.I. = [0.01, 3.58], *p* = 0.049.

#### Multidimensional Scaling of the Estimates

To fully understand the response patterns across all 32 sets and the variety of measures, we ran two multidimensional scaling (MDS) analyses, one based on the estimates and the other on the post-estimation ratings.

**FIGURE 8:**
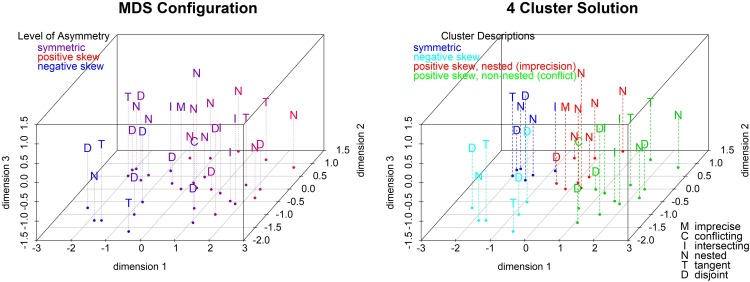
3-dimension MDS of all estimates by degree of asymmetry and with 4-cluster solution.

For the first solution, we calculated the Euclidian distance between the 32 mean profiles using five responses per profile: the best estimate, lower and upper bounds and lower and upper bounds of the 90% probability intervals. A three-dimensional solution (see **Figure 8**) yields the best fit (stress = 0.04 compared to 0.23 and 0.10 for 1- and 2-dimensional solutions). The left panel colors the conditions by the degree of asymmetry (from highest positive skew in red to highest negative skew in blue). Asymmetry correlates highly with the first two dimensions (see the scatterplot matrix in the Supplementary Materials): Positively skewed sets are high, and negatively skewed sets are low, on dimensions 1 and 2.

We performed a cluster analysis on the 3-dimensional solution to help interpret it. We used hierarchical clustering with Ward linkage because it is efficient and flexible to handle both chain-like and concentric clusters. Ward’s method is intuitively appealing since it minimizes the difference in sum of squares at each step in the algorithm. In the right panel of **Figure 8**, we impose the four-cluster solution that seems to be driven primarily by (a)symmetry and only to a lesser extent by overlap. One (cyan) cluster contains all negatively skewed sets (except one); a second (blue) cluster contains five (out of six) symmetric sets. The last two clusters contain primarily positively skewed sets. One (red) is almost entirely (7/8) nested sets including the imprecise set and a mix of five positively skewed and three symmetric sets. The other (green) is mostly (9/12) positively skewed sets and primarily the non-nested overlap categories including three quarters of the intersecting sets, the two positively skewed tangent sets, and five disjoint sets including the conflicting set. In summary, DMs’ estimates tend to vary as a function of the direction of skewness, and the nested sets lead to the most distinct estimates. This solution shows that asymmetry plays a large role in estimation, as expected, since M shifts within the projection set and the estimates shift correspondingly.

#### Multidimensional Scaling of the Post-estimation Ratings

We also calculated Euclidian distances between the 32 stimuli based on their seven comparative ratings: how (un-)ambiguous, (non-)conflicting, precise, credible, (likely to be) accurate, easy to reconcile, and complete each set was rated to be. The relevance of the source and type of uncertainty is apparent even for the one-dimensional solution with a stress of 0.27 (see Supplementary Materials). The conflicting case is at one end of this continuum and the imprecise set is at the other end with most hybrid sets located in-between (with only one exception at each end). The other clear result is that DMs rate disjoint and tangent sets as similar to the conflicting set (to the left end of the scale) and nested and intersecting sets as similar to the imprecise set (to the right end of the scale).

**FIGURE 9:**
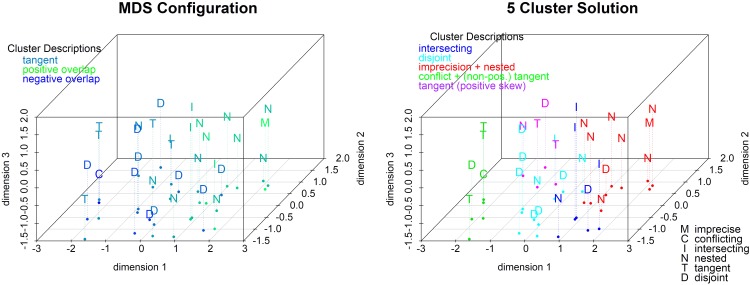
3-dimensional MDS of all ratings by degree of overlap and with 5-cluster solution.

The 3-dimensional solution is the best fitting solution (stress = 0.10) and is driven by both the degree and type of overlap. The left panel of **Figure 9** colors the conditions by the degree of overlap (from highest positive overlap in red to highest negative overlap in green). Overlap correlates highly with the first two dimensions (see the scatterplot matrix in the Supplementary Materials). Sets with a positive overlap are high, and sets with a negative overlap are low, on dimensions 1 and 2. We ran a cluster analysis using Wards linkage based on the distances from the 3-dimensional MDS solution. The five-cluster solution (see right panel of **Figure 9**) shows that imprecision forms a cluster with the nested sets which is close to the cluster with the intersecting sets. Conflict is on the other end of the plot and the disjoint cluster is nearby. The tangent sets are divided by direction of skewness, so positively skewed form one cluster, and non-positively skewed share a cluster with conflict. In summary, tangent sets are perceived as the most conflicting followed by disjoint, and nested sets are the most agreeing followed by intersecting.

## Discussion

As predicted, the best estimates were, essentially, compromises between the two expert forecasts’ and were unaffected by differences between the models. Most surprisingly, we found no effects of model uncertainty – neither the structural nor the judgmental component – on any of the dependent variables, but we found systematic and significant effects of source uncertainty. DMs responded to the most salient surface cues – in this case the values of the forecasts, including their relative agreement and precision, but they ignored more subtle, yet relevant, cues – the labels of the experts, models, and model parameters. These results are consistent with the system neglect hypothesis (Massey and Wu, 2005; Budescu and Yu, 2007).

There is a paradox in communicating CC information that as models get more complex, the public seems to become less sensitive to uncertainties in the models (e.g., Pidgeon and Fischhoff, 2011). Instead it appears that in domains involving deep uncertainty, such as CC, DMs are highly sensitive to the source of indeterminacy. Normatively, climate policy preferences should change according to the goal for uncertainties stemming from states of nature vs. differences between models (Drouet et al., 2015). As predicted, we observed a greater reduction in the judged range estimates (compared to the experts’ original forecasts) for *conflicting point* forecasts than for *agreeing imprecise interval* forecasts. DMs show a consistent dislike of, and aversion to, conflict and react more positively to communications that reflect imprecision: (1) Imprecision resulted in the greatest consistency with the expert forecasts; (2) DMs expressed higher confidence and preferred imprecise forecasts on characteristics ranging from credibility to completeness. This supports previous findings that conflict aversion is stronger than imprecision aversion (Smithson, 1999, 2015), and implies that there is a broader dimension, such as overall perception of uncertainty, that is driven by the degree of agreement between the forecasts.

The results confirm that DMs are not universal seekers of precision, but are rather sensitive to the nature, and features, of the decision environment. They expect a certain degree of imprecision or uncertainty in climate projections, and in line with the congruence principle (Budescu and Wallsten, 1995; Olson and Budescu, 1997; Du et al., 2011), they favor forecasts that seem to capture and reflect this imprecision. In fact, using bounded estimates to express uncertainty in climate projections leads to higher belief in and concern about CC since a high degree of uncertainty is expected (MacInnis et al., unpublished).

In the presence of hybrid projections that are both imprecise and conflicting, the DMs’ responses depend on the nature of the task. The joint effects of the two source uncertainties seem to lead to a compromise between the effects of imprecision and conflict for all judgmental tasks – confidence, willingness-to-pay for insurance and comparative ratings. However, we observed a combining pattern in estimation tasks – estimating the most likely value and range of possible values – where DMs displayed the least consistency with the experts in the hybrid condition. Response patterns do not reflect simple arithmetic on the endpoints. The relative agreement and configuration of the sets seem to promote differential weighting of the expert forecasts due to preferences and overall feeling about uncertainty.

These task-specific differences are consistent with the contingent weighting model (Tversky et al., 1988) and the subsequent task-goal hypothesis (Fischer et al., 1999) which state that task objectives influence response processes. We found differences in the processes used for *estimation* and *rating* tasks. In estimation tasks, DMs give range estimates closer to the endpoints under imprecision because those are the more prominent features, or focal points, while they give range estimates closer to the middle for the hybrid set since the common point (in Study 1), which is the midpoint of the set’s range, is the most prominent feature. In comparative ratings, DMs can see the hybrid set contains some features of conflict and some features of imprecision. Given that they are more averse to conflict than to imprecision (Smithson, 1999) they assign ratings that are more favorable than those of conflict, but less favorable than imprecision.

The focus of Study 2 was to develop a robust mapping of how conflict and imprecision are combined. Across the 30 different hybrid sets, reactions were a function of two key factors – structure of overlap and level of asymmetry. The results help explain why differences in cognitive processes and attention are used to respond to different goals. The size and direction of (a)symmetry of the two sets showed a stronger influence on quantitative estimates than their overlap, seemingly because asymmetry creates a tension between various measures of central tendency. Asymmetry highlights the deviation between the mean and median and makes it the prominent feature of the set. The structure and degree of overlap within a set had greater influence on preferential ratings seemingly because the (lack of) overlapping areas were the prominent set feature and altered the perception of (dis)agreement between experts. Sets with a positive overlap (nested and intersecting) were rated more similarly to imprecision indicating participants paid greater attention to the agreeing segment, and sets with a non-positive overlap (disjoint and tangent) were rated more similarly to conflict indicating participants paid greater attention to the distinct and disagreeing segments.

The two multidimensional scaling analyses confirm that estimates are driven by the *degree* of asymmetry and ratings are driven by the degree of overlap. Some pronounced response patterns within the key factors should be explored in further detail. First, heavy skewness caused the greatest bias from the set means because it creates the greatest discord between possible definitions of “center.” Our hybrid sets were confined to a common range (10–40 inches), so the most skewed sets had the largest discrepancy of interval widths between projections. The large degree of bias could indicate that participants weighted the experts based on the width of their forecast. A narrower, more precise, projection seems to be associated with greater credibility. Judges perceive a tradeoff between accuracy and the informativeness of others’ estimates where more narrow estimates are considered more informative, but less likely to be accurate (Yaniv and Foster, 1995). We extend these results to sets of multiple experts showing that experts providing narrower intervals are perceived as more credible and informative.

A large degree of overlap, whether an area of intersection (or positive DO) or space (negative DO), between the forecasts induced the greatest difference in ratings since it represents a larger unresolved region. A wide area of intersection suggests the experts agree, but the agreement is still imprecise. A wide area of disjointedness suggests the experts are far from agreement, and when the projections showed the most disagreement, participants rated the sets almost as conflicting and hard to resolve as pure conflict. This indicates that preferences are more complicated than following simple mathematical rules since sets with a large degree of overlap had the same range and same statistical center as corresponding sets with a small degree of overlap.

We did not observe differences based on belief in CC or political identification which, in principle, could have resulted in the discounting of projections that were inconsistent with one’s political affiliation. By design, the experts and models in this study were not individualized (expert A vs. B and model GCSX vs. ESGY). Thus, the observed patterns of preference cannot be attributed to prejudices about either. Social and political attributions of the experts’ motivation are a natural part of the assessment of their judgments, and it appears individuals make credibility judgments in this partisan domain even in the absence of identifying information. Moreover, recent evidence suggests attributions are a function of the educational and cognitive levels of the judges; those with lower education are more likely to attribute disagreement to incompetence, and those with higher education attribute disagreement to complexity and aleatory uncertainty (Dieckmann et al., 2015). Future work should consider the impact of these factors.

Unexpectedly we found the greatest shift away from the experts occurred when the set had a negative skew, especially for the best estimate and the lower bound. The shifting for all sets was toward lower values; best estimates were below the set mean and range estimates were narrower than the experts. This pattern is consistent with status quo bias and “system justification” – defending existing social systems – which is associated with discrediting CC (Feygina et al., 2010). Participants are less trusting of the worst-case projections, either because they do not believe the climate will continue to change at the current rate, or they tend to attribute “alarmist” motives to the forecasters who predict higher, and more threatening consequences from CC. Alternately, it implies that individuals intuit that the expected damage from CC has a specific shape that lower values are more likely than extremely high values (Lewandowsky et al., 2013). And, of course, these possibilities are not exclusive.

## Conclusion

In two studies, we have shown that perceptions of multiple climate projections are driven by the type and degree of disagreement between them, but the judges are insensitive to the differences between the models and how they were run. Moreover, judgmental reactions to the experts are driven by how two key factors – the structure of overlap and the level of asymmetry – interact with the task at hand. It appears that previously identified uncertainties stemming from multiple sources, conflict and imprecision, are special cases of overlap and asymmetry. Perceptions of agreement require intersection and balance. While, overly precise forecasts lead to a greater perception of disagreement among experts, and a greater likelihood of the public discrediting and misinterpreting information. Future studies should build on this work by exploring how the (mis)match between the judge and various experts alters perceptions of the evidence. Further, research should explore if overlap and asymmetry similarly impact perceptions of uncertainty in domains outside of CC.

## Ethics Statement

This study was carried out in accordance with the recommendations of Fordham University Institutional Review Board with written informed consent from all subjects. All subjects gave written informed consent in accordance with the Declaration of Helsinki. The protocol was approved by the Fordham University Institutional Review Board.

## Author Contributions

DMB: design, data collection, analysis, and writing. DVB: design, supervision, analysis plan, and editing.

## Conflict of Interest Statement

The authors declare that the research was conducted in the absence of any commercial or financial relationships that could be construed as a potential conflict of interest.

## Footnotes

1 Since there are only two values, *X*_min_ and *X*_max_, their mean, median and mid-range are identical.

2 Interestingly, we did not find significant association between the DMs’ responses with different levels of belief in climate change or political identification. Neither these factors, nor personal demographics were significant when included as covariates in the models.

3 We eliminated the quiz on the bidding procedure since we used a simple forced choice question.

4 All pair-wise comparisons based on Tukey–Kramer tests.

## References

[B1] Baillon A. Cabantous L. Wakker P. (2012). Aggregating imprecise or conflicting beliefs: an experimental investigation using modern ambiguity theories. *J. Risk Uncertain.* 44 115–147. 10.1007/s11166-012-9140-x

[B2] Becker G. M. DeGroot M. H. Marschak J. (1964). Measuring utility by a single-response sequential method. *Syst. Res. Behav. Sci.* 9 226–232. 10.1002/bs.3830090304 5888778

[B3] Bier V. M. Connell B. L. (1994). Ambiguity seeking in multi-attribute decisions: effects of optimism and message framing. *J. Behav. Decis. Making* 7 169–182. 10.1002/bdm.3960070303

[B4] Budescu D. V. Broomell S. B. Lempert R. J. Keller K. (2014a). Aided and unaided decisions with imprecise probabilities in the domain of losses. *EURO J. Decis. Process.* 2 31–62. 10.1007/s40070-013-0023-4

[B5] Budescu D. V. Kuhn K. M. Kramer K. M. Johnson T. R. (2002). Modeling certainty equivalents for imprecise gambles. *Organ. Behav. Hum. Decis. Process.* 88 748–768. 10.1016/S0749-5978(02)00014-6

[B6] Budescu D. V. Por H. H. Broomell S. B. Smithson M. (2014b). The interpretation of IPCC probabilistic statements around the world. *Nat. Clim. Change* 4 508–512. 10.1038/nclimate2194

[B7] Budescu D. V. Wallsten T. S. (1995). Processing linguistic probabilities: general principles and empirical evidence. *Psychol. Learn. Motiv.* 32 275–318. 10.1016/S0079-7421(08)60313-8

[B8] Budescu D. V. Yu H. T. (2007). Aggregation of opinions based on correlated cues and advisors. *J. Behav. Decis. Making* 20 153–177. 10.1002/bdm.547

[B9] Cabantous L. Hilton D. Kunreuther H. Michel-Kerjan E. (2011). Is imprecise knowledge better than conflicting expertise? Evidence from insurers’ decisions in the United States. *J. Risk Uncertain.* 42 211–232. 10.1007/s11166-011-9117-1

[B10] Casey J. T. Scholz J. T. (1991). Boundary effects of vague risk information on taxpayer decisions. *Organ. Behav. Hum. Decis. Process.* 50 360–394. 10.1016/0749-5978(91)90027-Q

[B11] Clemen R. T. (1989). Combining forecasts: a review and annotated bibliography. *Int. J. Forecast.* 5 559–583. 10.1016/0169-2070(89)90012-5

[B12] Cokely E. T. Feltz A. Ghazal S. Allan J. N. Petrova D. Garcia-Retamero R. (2018). “Decision making skill: from intelligence to numeracy and expertise,” in *Cambridge Handbook of Expertise and Expert Performance*, 2nd Edn, eds Ericsson K. A. Hoffman R. R. Kozbelt A. Williams A. M. (New York, NY: Cambridge University Press).

[B13] Dieckmann N. F. Johnson B. B. Gregory R. Mayorga M. Han P. K. Slovic P. (2017). Public perceptions of expert disagreement: bias and incompetence or a complex and random world? *Public Underst. Sci.* 26 325–338. 10.1177/0963662515603271 26346339

[B14] Dieckmann N. F. Mauro R. Slovic P. (2010). The effects of presenting imprecise probabilities in intelligence forecasts. *Risk Anal.* 30 987–1001. 10.1111/j.1539-6924.2010.01384.x 20409043

[B15] Dieckmann N. F. Peters E. Gregory R. (2015). At home on the range? Lay interpretations of numerical uncertainty ranges. *Risk Anal.* 35 1281–1295. 10.1111/risa.12358 25808952

[B16] Dieckmann N. F. Peters E. Gregory R. Tusler M. (2012). Making sense of uncertainty: advantages and disadvantages of providing an evaluative structure. *J. Risk Res.* 15 717–735. 10.1080/13669877.2012.666760

[B17] Ding D. Maibach E. W. Zhao X. Roser-Renouf C. Leiserowitz A. (2011). Support for climate policy and societal action are linked to perceptions about scientific agreement. *Nat. Clim. Change* 1 462–466. 10.1038/nclimate1295

[B18] Doran P. T. Zimmerman M. K. (2009). *Examining the Scientific Consensus on Climate Change, Eos, Transactions*, Vol. 90 Washington, DC: American Geophysical Union, 22–23 10.1029/2009EO030002

[B19] Drouet L. Bosetti V. Tavoni M. (2015). Selection of climate policies under the uncertainties in the Fifth Assessment Report of the IPCC. *Nat. Clim. Change* 5 937–940. 10.1038/nclimate2721

[B20] Du N. Budescu D. V. (2005). The effects of imprecise probabilities and outcomes in evaluating investment options. *Manag. Sci.* 51 1791–1803. 10.1287/mnsc.1050.0428

[B21] Du N. Budescu D. V. Shelly M. K. Omer T. C. (2011). The appeal of vague financial forecasts. *Organ. Behav. Hum. Decis. Process.* 114 179–189. 10.1016/j.obhdp.2010.10.005

[B22] Einhorn H. J. Hogarth R. M. (1985). Ambiguity and uncertainty in probabilistic inference. *Psychol. Rev.* 92 433–461. 10.1037/0033-295X.92.4.433

[B23] Einhorn H. J. Hogarth R. M. (1988). “Decision making under ambiguity: a note,” in *Risk, Decision and Rationality Theory and Decision Library (Series B: Mathematical and Statistical Methods)* Vol. 9 ed. Munier B. R. (Netherlands: Springer), 327–336.

[B24] Ellsberg D. (1961). Risk, ambiguity, and the Savage axioms. *Q. J. Econ.* 75 643–669. 10.2307/1884324

[B25] Erev I. Cohen B. L. (1990). Verbal versus numerical probabilities: efficiency, biases, and the preference paradox. *Organ. Behav. Hum. Decis. Process.* 45 1–18. 10.1016/0749-5978(90)90002-Q

[B26] Feygina I. Jost J. T. Goldsmith R. E. (2010). System justification, the denial of global warming, and the possibility of “system-sanctioned change”. *Pers. Soc. Psychol. Bull.* 36 326–338. 10.1177/0146167209351435 20008965

[B27] Fischer G. W. Carmon Z. Ariely D. Zauberman G. (1999). Goal-based construction of preferences: task goals and the prominence effect. *Manag. Sci.* 45 1057–1075. 10.1287/mnsc.45.8.1057

[B28] Fischhoff B. Davis A. L. (2014). Communicating scientific uncertainty. *Proc. Natl. Acad. Sci. U.S.A.* 111(Suppl. 4), 13664–13671. 10.1073/pnas.1317504111 25225390 PMC4183175

[B29] Frederick S. (2005). Cognitive reflection and decision making. *J. Econ. Perspect.* 19 25–42. 10.1257/089533005775196732

[B30] Galesic M. Kause A. Gaissmaier W. (2016). A sampling framework for uncertainty in individual environmental decisions. *Topics Cogn. Sci.* 8 242–258. 10.1111/tops.12172 26592358

[B31] González-Vallejo C. Bonazzi A. Shapiro A. J. (1996). Effects of vague probabilities and of vague payoffs on preference: a model comparison analysis. *J. Math. Psychol.* 40 130–140. 10.1006/jmps.1996.0012

[B32] Hammond K. R. (1996). *Human Judgment and Social Policy: Irreducible Uncertainty, Inevitable Error, Unavoidable Injustice.* New York, NY: Oxford University Press.

[B33] Heath Y. Gifford R. (2006). Free-market ideology and environmental degradation: the case of belief in global climate change. *Environ. Behav.* 38 48–71. 10.1177/0013916505277998

[B34] Heath C. Tversky A. (1991). Preference and belief: ambiguity and competence in choice under uncertainty. *J. Risk Uncertain.* 4 5–28. 10.1007/BF00057884

[B35] Hogarth R. M. Einhorn H. J. (1990). Venture theory: a model of decision weights. *Manag. Sci.* 36 780–803. 10.1287/mnsc.36.7.780

[B36] Holyoak K. J. Simon D. (1999). Bidirectional reasoning in decision making by constraint satisfaction. *J. Exp. Psychol. Gen.* 128 3–31. 10.1037/0096-3445.128.1.3 15149257

[B37] Joslyn S. L. LeClerc J. E. (2012). Uncertainty forecasts improve weather-related decisions and attenuate the effects of forecast error. *J. Exp. Psychol. Appl.* 18 126–140. 10.1037/a0025185 21875244

[B38] Kahan D. M. Jenkins-Smith H. Braman D. (2011). Cultural cognition of scientific consensus. *J. Risk Res.* 14 147–174. 10.1080/13669877.2010.511246

[B39] Kramer K. Budescu D. V. (2004). “Exploring Ellsberg’s paradox in vague-vague cases,” in *Experimental Business Research, 3*, eds Zwick R. Rapoport A. (Netherlands: Kluwer Academic Publishers), 131–154.

[B40] Kuhn K. M. (1997). Communicating uncertainty: framing effects on responses to vague probabilities. *Organ. Behav. Hum. Decis. Process.* 71 55–83. 10.1006/obhd.1997.2715

[B41] Kuhn K. M. Budescu D. V. (1996). The relative importance of probabilities, outcomes, and vagueness in hazard risk decisions. *Organ. Behav. Hum. Decis. Process.* 68 301–317. 10.1006/obhd.1996.0107

[B42] Kunda Z. (1990). The case for motivated reasoning. *Psychol. Bull.* 108 480–498. 10.1037/0033-2909.108.3.480 2270237

[B43] Larrick R. P. Soll J. B. (2006). Intuitions about combining opinions: misappreciation of the averaging principle. *Manag. Sci.* 52 111–127. 10.1287/mnsc.1050.0459

[B44] Lewandowsky S. Gignac G. E. Vaughan S. (2013). The pivotal role of perceived scientific consensus in acceptance of science. *Nat. Clim. Chang.* 3 399–404. 10.1038/nclimate1720

[B45] Lewandowsky S. Risbey J. S. Smithson M. Newell B. R. Hunter J. (2014). Scientific uncertainty and climate change: part I. Uncertainty and unabated emissions. *Clim. Change* 124 21–37. 10.1007/s10584-014-1082-7

[B46] Massey C. Wu G. (2005). Detecting regime shifts: the causes of under-and overreaction. *Manag. Sci.* 51 932–947. 10.1287/mnsc.1050.0386

[B47] Morgan M. G. Keith D. W. (1995). Subjective judgments by climate experts. *Environ. Sci. Technol.* 29 468A–476A. 10.1021/es00010a753 22667250

[B48] N.C. Coastal Resources Commission’s Science Panel on Coastal Hazards (2010). *North Carolina Sea-level Rise Assessment Report March 2010.* Available at: http://www.sealevel.info/NC_Sea-Level_Rise_Assessment_Report_2010--CRC_Science_Panel.pdf

[B49] Newell B. R. Pitman A. J. (2010). The psychology of global warming: improving the fit between the science and the message. *Bull. Am. Meteorol. Soc.* 91 1003–1014. 10.1175/2010BAMS2957.1

[B50] Olson M. J. Budescu D. V. (1997). Patterns of preference for numerical and verbal probabilities. *J. Behav. Decis. Making* 10 117–131. 10.1002/(SICI)1099-0771(199706)10:2<117::AID-BDM251>3.0.CO;2-7 17562000

[B51] Pidgeon N. Fischhoff B. (2011). The role of social and decision sciences in communicating uncertain climate risks. *Nat. Clim. Chang.* 1 35–41. 10.1038/nclimate1080

[B52] Russo J. E. Yong K. (2011). The distortion of information to support an emerging evaluation of risk. *J. Econom.* 162 132–139. 10.1016/j.jeconom.2010.07.004

[B53] Schwartz L. M. Woloshin S. Black W. C. Welch H. G. (1997). The role of numeracy in understanding the benefit of screening mammography. *Ann. Inter. Med.* 127 966–972. 10.7326/0003-4819-127-11-199712010-00003 9412301

[B54] Shanteau J. (2000). “Why do experts disagree,” in *Risk Behaviour and Risk Management in Business Life*, eds Green B. Cressy R. Delmar F. Eisenberg T. Howcraft B. Lewis M. (Dordrecht: Kluwer Academic Press), 186–196.

[B55] Siceloff B. (2014). *While the Seas Rise in the Outer Banks and Elsewhere in NC, Science Treads Water. The News & Observer.* Available at: http://www.newsobserver.com/news/politics-government/state-politics/article10298660.html

[B56] Smithson M. (1999). Conflict aversion: preference for ambiguity vs conflict in sources and evidence. *Organ. Behav. Hum. Decis. Process.* 79 179–198. 10.1006/obhd.1999.2844 10471360

[B57] Smithson M. (2015). Probability judgments under ambiguity and conflict. *Front. Psychol.* 6:674. 10.3389/fpsyg.2015.00674 26042081 PMC4438598

[B58] Tversky A. Sattath S. Slovic P. (1988). Contingent weighting in judgment and choice. *Psychol. Rev.* 95 371–384. 10.1037/0033-295X.95.3.371

[B59] Wallsten T. S. Budescu D. V. (1995). A review of human linguistic probability processing: general principles and empirical evidence. *Knowl. Eng. Rev.* 10 43–62. 10.1017/S0269888900007256

[B60] Wallsten T. S. Budescu D. V. Erev I. Diederich A. (1997). Evaluating and combining subjective probability estimates. *J. Behav. Decis. Making* 10 243–268. 10.1002/(SICI)1099-0771(199709)10:3<243::AID-BDM268>3.0.CO;2-M

[B61] Wallsten T. S. Budescu D. V. Zwick R. Kemp S. M. (1993). Preferences and reasons for communicating probabilistic information in verbal or numerical terms. *Bull. Psychon. Soc.* 31 135–138. 10.3758/BF03334162

[B62] Weller J. A. Dieckmann N. F. Tusler M. Mertz C. K. Burns W. J. Peters E. (2013). Development and testing of an abbreviated numeracy scale: a Rasch analysis approach. *J. Behav. Decis. Making* 26 198–212. 10.1002/bdm.1751 PMC7161838 32313367

[B63] Yaniv I. Foster D. P. (1995). Graininess of judgment under uncertainty: an accuracy-informativeness trade-off. *J. Exp. Psychol. Gen.* 124 424–432. 10.1037/0096-3445.124.4.424

[B64] Yaniv I. Milyavsky M. (2007). Using advice from multiple sources to revise and improve judgments. *Organ. Behav. Hum. Decis. Process.* 103 104–120. 10.1016/j.obhdp.2006.05.006

[B65] Zehr S. C. (2016). Public representations of scientific uncertainty about global climate change. *Public Underst. Sci.* 9 85–103. 10.1088/0963-6625/9/2/301

[B66] Zickfeld K. Morgan M. G. Frame D. J. Keith D. W. (2010). Expert judgments about transient climate response to alternative future trajectories of radiative forcing. *Proc. Natl. Acad. Sci. U.S.A.* 107 12451–12456. 10.1073/pnas.0908906107 20616045 PMC2906595

