# Supplementary material for: The Role of Type and Source of Uncertainty on the Processing of Climate Models Projections
Source: Front Psychol. 2018 Mar 27;9:403. doi: 10.3389/fpsyg.2018.00403 (PMC5881250; doi:10.3389/fpsyg.2018.00403)

**Supplementary Materials**

**INSTRUCTIONS**

**Background Information**

 A significant body of scientific evidence suggests that the Earth’s climate is changing and that its temperature is increasing. This trend has accelerated in the latter half of the twentieth century and continues today.  The oceans are expanding because they are warmer, and many glaciers are melting, causing sea levels to rise. Evaporation from warmer oceans increases the amount of moisture in the atmosphere and thus the amount of rain and snow in some places.  In general, global temperature tends to increase with the amount of carbon dioxide in the atmosphere, which has been increasing since industrialization.  Human use of fossil fuels such as coal, gasoline, and natural gas contribute to emissions of carbon dioxide and are correlated with global climate change.

**Why Climate Change Matters**

 Scientists and policy-makers are concerned that these changes will impact human, animal and plant life throughout the planet. For instance, rising sea levels will make some areas uninhabitable and increase the frequency of flooding. Changing patterns of rainfall and snowfall can lead to flooding in certain regions but droughts in others. These impacts, along with increasing temperatures, can alter the habitats of many species causing some species to move to new, colder areas where they can find their food.  In addition, the infrastructure in many American cities may prove poorly-suited for the new climate conditions and may require major re-investments.

While a significant body of scientific evidence suggests that globally average temperatures are increasing and that sea levels are rising due to human interference, scientists disagree about many important details of these changes.  For example, they disagree about how quickly the climate is changing and how specific regions will be affected.

**Earth System Models**

 To help understand these future changes, scientists often use complicated models called Earth System Models (EaSMs).  These models provide projections of future climate based on current measurements and “state of the art” science. These models simulate the Earth’s climate based on the behavior of the factors believed to influence it, such as atmospheric gases and chemicals, atmospheric and oceanic circulation patterns, carbon cycles, sea and ice levels, ash and dust, human factors, and many others.  The impact of each factor is determined by a series of simulated “experiments” typically comparing past measurements on key variables with climate conditions in the following time periods.  Researchers run these simulations within a specified, future time period (making projections for the next 10 years, the next 50 years, etc.)

 To understand the potential consequences of climate change, scientists combine EaSMs with models of the specific regions and sectors that might be affected.  For example, scientists can study

- What are the effects of changes in temperature and precipitation on agriculture in specific geographic regions?
- How will changes in climate alter the natural habitats of certain plants and animals?
- How will climate change affect tropical storms (for example, will hurricanes and tornadoes become stronger and/or more frequent)?

**Why EaSM Projections are Uncertain**

 Different models have been developed by scientists all over the world. They differ in the particular variables included, assumptions about the rules that govern their behavior, the level of detail used to describe various physical processes, and how precisely they measure various factors.  Thus, although the models are often in agreement, sometimes they make different projections.

 In some cases, it can take a year or more to obtain results from an EaSM.  Each projection relies on specific assumptions about the behavior of certain key factors in the future (e.g., the rate of greenhouse gas emissions in the next 25 years), so different runs of any given model may yield different projections based on the assumptions made.  Because of the time and cost required for each projection, scientists are limited in the number of variations (meaning changes in their initial assumptions) they can consider.  Thus, EaSM projections always come with a degree of uncertainty.

**The Current Study**

 In this study we will present you with three different sets of projections generated by various climate change models (such as an EaSM) with different initial conditions. The projections relate to a variety of potential consequences associated with global climate change (such as changes in water level in the oceans and their impact on the planning of US ports, or changes in precipitation patterns and their impact of water planning).  In each case, you will receive multiple predictions, and you will be asked to answer questions and make some judgments and predictions based on, and about, them.

 First we would like you to answer a brief questionnaire that consists of various statements about the relationship between humans, the environment and the earth’s climate.  **You will earn $10 for completing this task.  You can use this money later, in the first set of decisions.**

**Scenario Presented to the DMs**

Coastal experts in southern California have run projections of sea levels between Los Angeles and San Diego under various models of climate change, and they have determined that because of warming water and melting icecaps over the next fifty years, sea levels will increase substantially. Although there is agreement about this outcome, there is some disagreement among models and the experts using them on the timeline and severity of the problem. Coastal authorities are considering improvements to the ports in these communities, so they can continue to function.  A primary issue is the cost of raising the ports’ docks, bridges, and other facilities so they can avoid flooding. The authorities cannot afford to rebuild more than once, and they anticipate a substantial increase in cost for each additional inch in height:

Two experts (call them A and B) ran [one or two models (GCSX or ESGY)] with [one set of common values or with each expert using different values] reflecting the experts’ beliefs about the key parameters.  They made the following predictions based on these runs:

Scientist A used model GCSX to project an increase in sea level of [source uncertainty] inches over the next 50 years.

Scientist B used model [GCSX or ESGY] to project an increase in sea level of [source uncertainty] inches over the next 50 years.

GCSX = Global Circulation Simulation model - version X

ESGY = Earth System Generation model - version Y

Note: Differences between projections may reflect the experts' uncertainty about the values of the key parameters.

Based on this information, what is your estimate of the number of inches that sea level will increase in southern California over the next 50 years?

Table S1

*Belief in Climate Change Scales for Study 1*

For each statement, please indicate whether you STRONGLY DISAGREE, MILDLY DISAGREE, are UNSURE, MILDLY AGREE, or STRONGLY AGREE with it.

| Set 1 | Set 2 | Scale | Variable | Item |
| --- | --- | --- | --- | --- |
| 1 | 1 | BGW | Y | [GW/CC] is occurring now. |
| 1 |  | BGW | Y | I am quite sure that [GW/CC] is occurring now. |
|  | 1 | BGW | Y | I do not believe that [GW/CC] is real. |
| 1 | 1 | PE | Y | I have already noticed some signs of [GW/CC]. |
| 1 |  | PE | N | It seems to me that temperature is warmer now than in years before. |
|  | 1 | PE | N | It seems to me that weather patterns have changed compared to when I was a child. |
| 1 |  | PCA | Y | The main causes of [GW/CC] are human activities. |
|  | 1 | PCA | Y | [GW/CC] is merely a natural fluctuation, not caused by human activity. |
| 1 | 1 | PCA | Y | I am quite sure that human activities are responsible for [GW/CC]. |
| 1 | 1 | PCO | Y | The consequences of [GW/CC] will be harmful for the environment. |
| 1 |  | PCO | Y | [GW/CC] will bring about some serious negative consequences. |
|  | 1 | PCO | Y | The consequences of [GW/CC] will be more positive than negative overall. |
| 1 | 1 | SE | Y | There are simple things that we can do that will have a meaningful effect to alleviate the negative effects of [GW/CC]. |
| 1 |  | SE | Y | I believe that little things we can do will make a difference to alleviate the negative effects of [GW/CC]. |
|  | 1 | SE | Y | Even if we try to do something about [GW/CC], I doubt if it will make any difference. |
| 1 | 1 | IAG | Y | I plan to take some actions to stop [GW/CC]. |
|  | 1 | IAG | Y | I will make some efforts to mitigate the negative effects of [GW/CC]. |
| 1 |  | IAG | Y | I intend to take concrete steps to do something to mitigate the negative effects of [GW/CC]. |

Labels: GW = Global warming, CC = Climate change. Labels were assigned randomly for each set.
Scales: BGW = belief in global warming, PE = personal experience, PCA = perception of causes, PCO = perception of consequences, SE = self-efficacy, IAG = intentions to act (general).

Table S2

*Belief in Climate Change Scales for Study 2*

For each statement, please indicate whether you STRONGLY DISAGREE, MILDLY DISAGREE, are UNSURE, MILDLY AGREE, or STRONGLY AGREE with it.

| Set 1 | Set 2 | Scale | Variable | Item |
| --- | --- | --- | --- | --- |
| 1 |  | BGW | Y | [GW/CC] is occurring now. |
|  | 1 | BGW | Y | I am quite sure that [GW/CC] is occurring now. |
| 1 |  | PE | Y | I have already noticed some signs of [GW/CC]. |
|  | 1 | PE | N | It seems to me that temperature is warmer now than in years before. |
|  | 1 | PCA | Y | The main causes of [GW/CC] are human activities. |
| 1 |  | PCA | Y | I am quite sure that human activities are responsible for [GW/CC]. |
| 1 |  | PCO | Y | The consequences of [GW/CC] will be harmful for the environment. |
|  | 1 | PCO | Y | [GW/CC] will bring about some serious negative consequences. |
| 1 |  | SE | Y | There are simple things that we can do that will have a meaningful effect to alleviate the negative effects of [GW/CC]. |
|  | 1 | SE | Y | I believe that little things we can do will make a difference to alleviate the negative effects of [GW/CC]. |
| 1 |  | IAG | Y | I plan to take some actions to stop [GW/CC]. |
|  | 1 | IAG | Y | I intend to take concrete steps to do something to mitigate the negative effects of [GW/CC]. |
| 1 | 1 | KNO | Y | I am well-informed about [GW/CC] |

KNO=knowledge

**Numeracy Scale**

Adapted from Weller et al., 2015

1. In the BIG BUCKS LOTTERY, the chances of winning a $10.00 prize are 1%. What is your best guess about how many people would win a $10.00 prize if 1,000 people each buy a single ticket from BIG BUCKS?

2. In the ACME PUBLISHING SWEEPSTAKES, the chance of winning a car is 1 in 1,000. What percent of tickets of ACME PUBLISHING SWEEPSTAKES win a car?

3. A bat and a ball cost $1.10 in total. The bat costs $1.00 more than the ball. How much does the ball cost?

 4. In a lake, there is a patch of lilypads. Every day, the patch doubles in size. If it takes 48 days for the patch to cover the entire lake, how long would it take for the patch to cover half of the lake?

The following two items were included in Study 1, and excluded from Study 2.
Item 5 had a similar item difficulty to item 1, and item 6 was too easy.

5. If the chance of getting a disease is 10%, how many people would be expected to get the disease? Out of 1000?

6. If the chance of getting a disease is 20 out of 100, this would be the same as having a ____% chance of getting the disease.

Table S2 *Effects for All Outcomes (Study 2)*

| Outcome (F) | Source df = 2/928 | Overlap df = 3/929 | Asymmetry df = 1/929 | Overlap X Asymmetry df = 3/929 |
| --- | --- | --- | --- | --- |
| Estimate (Deviation from Mean) | 10.9 |  |  |  |
| Confidence (Est) | 3.47 |  |  |  |
| Range | 9.36 | 4.40†/3.04‡ |  | 2.78 |
| Lower Bound | 13.85 | 2.85 | 5.35 |  |
| Upper Bound | 9.42 | 4.21† | 8.98 |  |
| Insurance Bid |  |  |  |  |
| Confidence Interval | 3.45 |  | 6.12 |  |

The Source column describes MANOVA results.
Overlap, asymmetry, their interaction only describe models of hybrid forecast sets only; †Only applies to hybrid vs. imprecision. ‡Only applies to hybrid vs. conflict.
Source df_den_ = 927 for confidence and 915 for CI.
Asymmetry df_den_ = 929 for CI.

Figure S1
*Scatterplot Matrix of 3 Scaling Dimensions of the Estimates with Key Factors (Study 2)* *
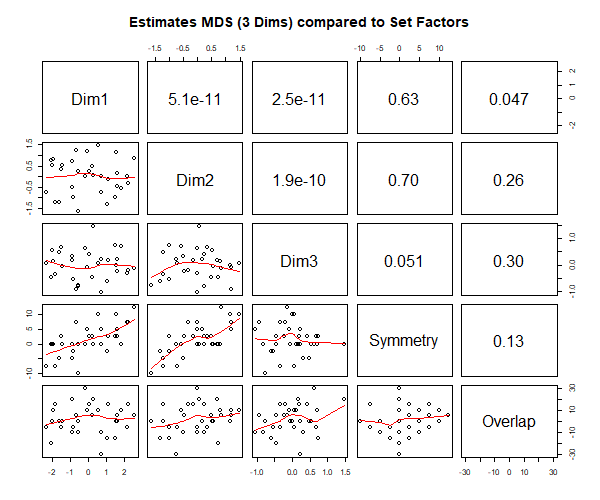
*

Figure S2
*Uni-Dimensional Scaling of All Ratings Labeled by Type of Overlap and Colored by Skewness (Study 2)*


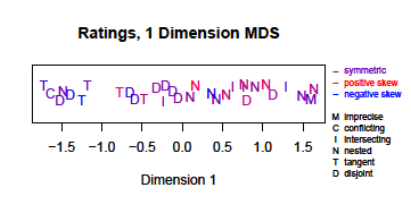


Figure S3
*Scatterplot Matrix of 3 Scaling Dimensions of the Ratings with Key Set Factors (Study 2)*

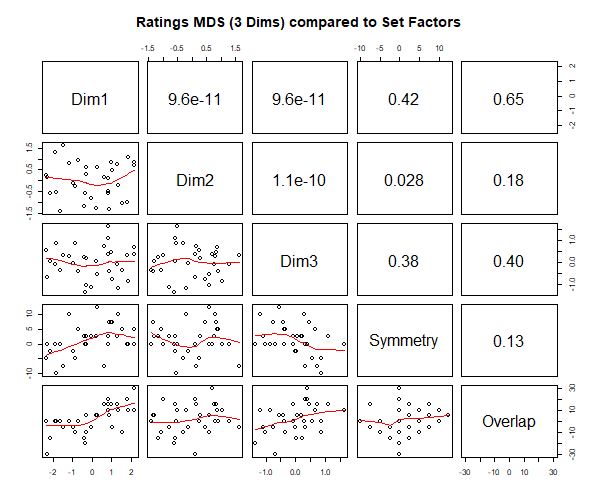

Supplement: Supplementary file 1 [file Data_Sheet_1.docx]
